# Perceptions and experiences of a manual therapy trial: a qualitative study of people with moderate to severe COPD

**DOI:** 10.1186/s12998-021-00387-0

**Published:** 2021-07-27

**Authors:** Danielle A. Baxter, Johannah L. Shergis, Catherine J. Hill, Christopher Worsnop, Meaghan E. Coyle

**Affiliations:** 1 School of Health and Biomedical Sciences, RMIT University, Victoria Bundoora, Australia; 2 Department of Physiotherapy, Austin Health, Heidelberg, Victoria Australia; 3 Institute for Breathing and Sleep, Austin Health, Heidelberg, Victoria Australia

**Keywords:** COPD, Complementary medicine, Manual therapy, Participant experiences, Preferences, Motivations

## Abstract

**Background:**

Chronic obstructive pulmonary disease (COPD) presents with physical, emotional and social difficulties that affect quality of life. Multimodal management includes both pharmacological and non-pharmacological strategies, and pulmonary rehabilitation (PR) plays an important role. Recent research has suggested that manual therapies may improve perceptions of dyspnea for people with COPD.

**Methods:**

Focus group interviews were conducted as part of a mixed methods study to assess the feasibility of implementing a manual therapy technique—muscle energy technique (MET)—as an adjunct to PR for people with moderate to severe COPD. Focus group interviews were conducted to examine trial participants views of the intervention and the trial design. A thematic analysis was undertaken to explore the data.

**Results:**

Twelve participants with moderate to severe COPD participated in three focus groups. Participants were motivated to participate in the trial to be proactive about their health. They perceived MET to be a gentle, comfortable form of stretching that allowed them to ‘breathe easier’ and prepared them for PR. A small number of participants reported mild muscular discomfort during MET, but this was short-lasting and was not bothersome. Participants enjoyed the one-on-one contact with researchers and learned more about their breathing while performing spirometric testing. Most participants wanted longer and more frequent MET sessions, and some requested ‘homework’ stretching exercises.

**Conclusions:**

The findings of this study show that a manual therapy intervention was received well by participants in a clinical trial setting. A small number of participants reported mild musculoskeletal discomfort in relation to the MET treatment. Participant preferences for additional and longer treatment sessions should be carefully considered against available resources in future clinical trials.

**Trial registration:**

ANZCTR, ACTRN12618000801213. Registered 11 May 2018 - Retrospectively registered. http://www.anzctr.org.au/Trial/Registration/TrialReview.aspx?id=374643&isReview=true

**Supplementary Information:**

The online version contains supplementary material available at 10.1186/s12998-021-00387-0.

## Introduction

People with chronic obstructive pulmonary disease (COPD) have varying degrees of physical, emotional and social difficulties, which often increase as the disease progresses [1, 2]. Consequently, management of COPD is multimodal and combines pharmacological and non-pharmacological strategies [3]. Pulmonary rehabilitation (PR) is a widely used, non-pharmacological treatment, and there is strong evidence that it improves exercise capacity and self-rated dyspnea levels [4]. However, there are several barriers to attendance and adherence to PR for people with COPD, such as deterioration in health [5] and degree of breathlessness [6].

The considerable variability of symptoms between individuals with COPD [7, 8] may affect adherence and uptake of non-pharmacological management. Conversely, factors that increase attendance [9] to non-pharmacological management—such as PR—include healthcare professionals, motivation and encouragement and seeing benefits [5]. Employing multi-faceted interventions that provide individualized consultation approaches [10] and strengthen patient-practitioner relationships [11] have shown favorable results in clinical outcomes. The integration of a complementary and alternative medicine (CAM) intervention alongside PR may be one way to increase uptake and adherence to PR.

Previous studies have evaluated the use of manual therapy as an adjunct to PR or a structured exercise program [12–14], and systematic reviews have sought to quantify the effectiveness of manual therapy [15, 16] and specific techniques (e.g., spinal manipulation) [17], for COPD. Diversity in the interventions and outcome measures have produced conflicting results. Most studies have focused on clinical outcomes; however, two studies that explored the immediate effect of osteopathic techniques [18, 19] found that most participants enjoyed the osteopathic treatment and felt they could breathe better following treatment, even though there were minimal changes in lung function.

Given there is a growing awareness of the importance of the patients’ views and management preferences in providing health care [20], it is important to capture rich qualitative data. This study aimed to examine participants’ experiences and perceptions of receiving a manual therapy intervention—muscle energy technique (MET)—as an adjunct to PR in a clinical study setting.

## Methods 

This qualitative research was a part of a mixed methods feasibility study of MET as an adjunct to PR for people with moderate to severe COPD. MET is a hands-on technique that is used by many allied health practitioners—including osteopaths [21–23], chiropractors [24] and physical therapists [25]—to treat musculoskeletal complaints. Participants underwent 8 weeks of PR and MET and were assessed 4 weeks after the end of treatment. The PR component was an hour session in the gym, conducted twice weekly for 8 weeks. The MET component involved 15 min of tailored muscle stretches to the chest and back/neck regions performed by a registered osteopath just prior to PR for six out of the 8 weeks. An example of a MET procedure involved the practitioner positioning the patient in a particular position (e.g., trunk rotated to the left in the seated position), the participant contracting their paravertebral muscles to push in the opposite direction (e.g., trunk rotation to the right) while the practitioner applies a distinct counterforce against the participant movement for 3–5 s. Following this, the participant relaxes, and the practitioner places the participant into further trunk rotation to the left. This is repeated 3–5 times. See Additional File 1 for examples of treatment positions used during the trial.

A qualitative study was incorporated to provide a deeper understanding of participants views and experiences of participating in a non-controlled clinical study. Focus groups were considered the most appropriate design, as the group dynamics allow for deeper insights from participants. Furthermore, the interpersonal communication and social interaction may encourage contributions from people in the group who are reluctant to be interviewed by themselves, or who feel they do not have anything to say about their experience [26].

Participants who had completed at least two MET sessions and the clinical study exit interview were eligible. The number of focus groups conducted was determined from the sample size of the clinical study (*n* = 33), the eligible sample size for the focus groups (*n* = 27) and the optimum number of people recommended for focus groups—between 6 and 10 participants in each focus group [27, 28]. Clinical study participants were sent an invitation letter and patient informed consent form by mail or were informed about the focus groups at their final study visit. Written informed consent was obtained prior to the focus group. The study received ethical approval from the Austin Hospital Human Research Ethics Committee (HREC/16/Austin/474) and was also registered with Australian New Zealand Clinical Trials Registry (ACTRN12618000801213). Results are reported following the Consolidated Criteria for Reporting Qualitative Research checklist (see Additional file 2).

### Group interviews

Focus groups were conducted in a meeting room at the physiotherapy department where participants had attended the PR and MET sessions. A semi-structured interview guide (with follow-up probing questions) was used. The interview guide was developed to explore participant perceptions of study implementation, the participant experience of the trial—with a focus on the MET aspect—and aspects of the trial design that participants would change if there was a future study (see Additional file 3). The interview guide was not pilot tested, due to the feasibility nature of the study design. The interview guide was reviewed after each focus group to determine whether any changes were required; no such changes were made.

Focus groups were facilitated by one researcher (MC) while a second researcher (DB) took notes; discussion lasted between 30 and 60 min. Both researchers who facilitated the focus groups (MC and DB) are registered allied health professionals with experience in communication with patients, which provided a good basis for ensuring clinical relevance in the interpretation. All focus groups were audio recorded and were uploaded to a password-protected computer for storage and preparation for transcription. In return for their time, participants were offered free parking within the hospital carpark for ease of access, and they were provided with morning tea.

Recordings were transcribed verbatim and were de-identified. True internal anonymity was unachievable due to the primary qualitative researchers (DB and MC) having access to the participant data and having met them in person. Potentially identifying information in the transcripts were replaced to anonymize the qualitative study participants to an external audience. Member checking (i.e., returning transcripts to participants for verification) was not used, as studies have shown that this process can result in revisions that no longer reflect the interaction [29]. Triangulation of results from the clinical study exit interview (data not presented) was used to promote objectivity and increase validity of the findings. Finally, the researchers (DB and MC) organized frequent peer debriefing sessions to discuss and interpret the data.

### Data analysis

A qualitative descriptive approach was used to analyze the data. Thematic analysis used the following steps: familiarizing with data, generating initial codes, searching for themes, reviewing themes, defining and naming themes, and producing the report [30]. Familiarity with the data was obtained by having the same researcher (DB) transcribe the audio from the focus groups. Further preparation of the raw data files involved formatting the information to be compatible with NVivo 12 Pro, the software used to facilitate data analysis [31].

The same researcher (DB) initially read through the data without taking any notes, and then re-read it several times to gain an understanding of the data. Inductive (or in vivo) coding was used to reflect the participants’ words and maintain their meaning. This approach also means that the themes are closely linked to the data, and not driven by the researcher’s underlying theoretical assumptions [30]. Transcripts and codes were reviewed by a second researcher (MC), and codes were categorized into potential themes. The final patterns, codes and themes were the result of an iterative process of reviewing the themes and cross-checking in relation to the codes and the entire data set. Consensus was reached on the final set of codes, themes and subthemes, and quotations relevant to each theme were selected.

## Results

Twelve of the 27 clinical study participants (44.4%) who completed the exit interview of the clinical study attended one of three separate focus groups, held between April 2019 and December 2019. The characteristics of participants are summarized in Table 1.

**Table 1:** Characteristics of participants

Characteristic	Focus group 1 (*n* = 3)	Focus group 2 (*n* = 5)	Focus group 3 (*n* = 4)
Gender			
Male	3	4	2
Female	0	1	2
Age (range, years)	78–88	74–78	61–76
COPD severity^a^			
Moderate	1	3	3
Severe	2	2	1

^a^COPD severity measured in terms of Global Initiative for COPD (GOLD) guidelines [3]

There were no drop-outs during the focus groups. The differences between focus group participants and non-participants were analyzed at baseline and end-of-treatment for three outcome measures: modified Medical Research Council dyspnea index (mMRC), BODE index and COPD Assessment Test (CAT). These outcome measures were chosen as they capture the individual’s own symptomatic evaluation of their experience of COPD, which is relevant to the present qualitative study. Multivariate effects between focus group participants and non-focus group participants showed no statistical difference (*p* > 0.05) for all three outcome measures (data not presented).

### Themes

Thematic analysis of three participant focus groups revealed five broad themes: 1) ‘Motivations for participating’, 2) ‘Expectations of treatment’, 3) ‘Experiences of MET’, 4) ‘Perceptions of participating in the trial’ and 5) ‘Perceptions of the trial design’. The themes presented in the following section are shown in Table 2.

**Table 2:** Themes and subthemes

Themes	Subthemes
Motivations for participating	–
Expectations of treatment	Prior experience
	Knowing what to expect
Experiences of muscle energy technique	Experiences during muscle energy technique
	Relaxed and easy breathing
	Warm up for pulmonary rehabilitation
	Side effects
Perceptions of participating in the trial	Personnel
	Getting breathing right
Perceptions of the trial design	Treatment features
	Wanting to do more

#### Motivations for participating

Several factors influenced participants’ motivations, including their experience of living with COPD, the need to be more proactive about their health and the benefits of exercise. Interestingly, in all three focus groups, participants described their experiences of living with COPD and some of the things they had to discontinue due to their symptoms, such as being able to stretch by themselves. One man lamented that he was never able to get back on the tennis court because he just ‘couldn’t get enough breath’ (male, focus group 1). Others spoke about the anxiety they experience with their breathlessness:

You’ve already got COPD, you got anxiety about how you breathe and, you know, your breathing is running out. (Female, focus group 3).

Some participants showed insight into their disease and acknowledged factors that were within their control, despite not always choosing to help themselves. The following interaction between participants highlighted the contrasting attitudes to living with COPD:

I believe once your lungs are damaged your lung function remains, it’s not going to improve. It’s how you deal with it. (Female, focus group 3).

True. And I’m … I don’t really deal with it all that well. I don’t help meself [sic], you know. I s’pose [sic] a bit more exercise has gotta [sic] do something. (Male, focus group 3).

Participants also described their initial thoughts about the trial, and the perceived potential benefits from partaking in the study. Some participants expressed the general attitude that participating would only help them. One male went into further detail about why he thought participating would be worthwhile, and discussed that he was intrigued to find out about chest exercises:

I thought, well it’s gotta [sic] be good. You know? It’s exercise; and its exercise for where you don’t normally get specific exercises. You can get exercises for your legs and your back and your arms, but this was your chest … and so, I thought, it’s really gotta [sic] be worthwhile. (Male, focus group 1).

Several participants expressed altruistic motivations for participating in the study. One participant shared that she wanted to support someone doing their PhD, as she had recently had a family member obtain their PhD qualification. Others were motivated to help other people with COPD. One person articulated her strong feelings towards participating in research projects in general to help other people:

Like I always believe … if we don’t involve ourselves, you’re never gonna [sic] find out how it works and whether its gonna [sic] benefit in the long run. (Female. Focus group 3).

In addition to wanting to help other people with COPD, one male also emphasized a desire to ‘give back’ to the medical profession as his primary reason to participate:

On my behalf, the medical profession has done a lot for me. And apart from the benefits or non-benefits I may have gotten, or may not have gotten out of this, I was only too glad to give something back. (Male, focus group 2).

#### Expectations of treatment

This theme describes the participants’ perceptions of what they were expecting with the MET treatment. The subthemes related to their prior experience of consulting an osteopath and the information the participants received prior to the participation in the study.

##### Prior experience

In general, most participants had not seen an osteopath prior to the clinical study, nor had they received or heard of MET from any allied health practitioner. Some people had previously seen a massage therapist or a physiotherapist and clarified that it was for back or shoulder pain, not for their breathing. Of the few that were familiar with osteopathy, one person saw an osteopath regularly and explained his prior experience:

I must admit what I do at the osteopath is not so much for the lungs. It’s for the shoulders and the lower back. (Male, focus group 2).

##### Knowing what to expect

Although most people had no prior experience with MET, all participants felt that they received enough information about the study. There were several participants who did not know what to expect because of unfamiliarity with the technique:

I think most of us probably didn’t know what to expect ‘cos [sic] we’d never heard about it. (Female, focus group 3).

After receiving information about the study, several participants mentioned that they felt participating in the study was worthwhile. One person reasoned that MET would not be a strenuous form of activity after reading the information form. Some participants also wondered whether it would cause muscular or breathing discomfort. One participant likened the potential musculoskeletal pain to a feeling he would get after sport when he was younger:

I thought I would’ve, might’ve got stiffness. Because, ah, when you were young, and you might remember (*motioning to another participant*)? You’d go through the first week of footy training or some sort of training. And, your calves, and you could hardly move, and you thought ‘oh God almighty’, you know? So, I knew what [it] was and I thought, well if I’m gonna [sic] get stretched like this, I might get some discomfort. But I didn’t, I didn’t get any. (Male, focus group 1).

#### Experiences of muscle energy technique

This theme included four subthemes identified that were related to how participants felt during the MET session, any subjective changes in their physical being after MET, how MET prepared the participant for PR and whether they experienced any negative effects associated with MET.

##### Experiences during muscle energy technique

Across all three focus groups, there were several participants who described the MET session as relaxing and gentle. One participant described the musculoskeletal effects that he experienced during the MET session:

It was more than comfortable. I think it was relaxing. Firstly, it took away a lot of the aches that I seem to constantly have with my chest, my back etcetera. Just that amount of twisting, and that extra two or three twists. It made me feel very comfortable. I was terribly relaxed. In fact, I was afraid sometimes that I would, sort of, doze off. (Male, focus group 2).

In addition to the MET feeling like a gentle and easy form of treatment, participants also expressed their enjoyment in being stretched. One participant described the experience of being stretched as ‘pleasurable’ because they felt COPD stops them from stretching themselves. Another person spoke about why they thought the stretching felt good:

It was hard ‘cos [sic] you’re trying to push that muscle further with the way (lead researcher) was doing the treatment. Like one step is one stretch and then she goes a bit further and then a bit further. It feels good on your body. Definitely feels good on your body to be able to do that. (Female, focus group 2).

##### Relaxed and easy breathing

Several participants also described some positive effects immediately after the MET session. Participants reported a sense of ‘relaxation’ and a ‘looseness’ in the muscles after receiving MET that was beneficial when they went into the PR session:

I found it helped me with exercises … and, ah, by the time I come in [to PR] at lunch time, well, I was walking alright and didn’t have the aches and pains in the back and shoulders. I certainly found that [MET] loosened my body up or muscles up to do the exercises and that feeling remained with me till the end of the day. (Male, focus group 2).

Some participants also described a feeling of being able to breathe easier, and that they felt like they had ‘more room to breathe’ immediately after the MET session. One participant described the longer term benefits that she felt were due to MET:

I found my breathing looser and free. So much so, that I am going to organise to go to a physiotherapist. At least fortnightly. Just to pursue any benefit, or the option of benefits. Um, because what [lead researcher] did was, and I’ve discussed this with [lead researcher], it’s worth, it’s worth proceeding. (Female, focus group 2).

In contrast, some participants did not find any change in their breathing. One man reported that he had not noticed any difference in his breathing but added that he thought it might be because he saw an osteopath regularly prior to the study. Another man shared the following sentiments:

Yeah, I enjoyed, you know, what I went through there, but I think at the end of it all … ah it still hasn’t helped me [sic] breathing. (Male, focus group 3).

##### Warm up for pulmonary rehabilitation

The addition of MET to PR was discussed in relation to timing and the effects of completing both components of the intervention on the same day. Several participants described the MET as being complementary to PR, in that they felt more relaxed and better able to work on the exercise machines after MET. Furthermore, one of the most commonly described benefits of completing MET prior to PR was that participants felt like it was a ‘warm up’ for the exercises in the gym:

Actually, I think it was a pretty good warm up for coming in here and doing the other exercises and stuff, especially the bike and, you know … and the treadmill. (Male, focus group 3).

Interestingly, one participant noted how this perceived ‘warm up’ also impacted her motivations during the PR session:

I think that’s what actually inspires you to do the next step as well … Oh yeah, I’ve been stretched so now I can go and do my physical exercises a bit better because I’ve already done that warm up. (Female, focus group 3).

Conversely, there was one participant who did not like the timing of MET prior to the PR session, as he felt that it somewhat reversed the effects of MET:

For me, because I came straight in from having that done by [lead researcher] to do physio [PR] … by the time I finished physio, approximately about an hour, everything had been undone. (Male, focus group 2).

##### Side effects

A small number of participants (2 out of 12, 17%) mentioned that they experienced some mild muscular discomfort during a MET session. Musculoskeletal discomfort is commonly reported after manual therapy and participants were advised of this prior to entering the study. Participants who experienced discomfort described it as transient, self-resolving and were generally unperturbed by it. One participant shared his experience:

[I] didn’t have any pain, like you were saying, but occasionally I felt like my muscles around my shoulders were worked out a little bit more … but as you said, the pain would go away like within that day. (Male, focus group 3).

Some participants agreed with the philosophy of ‘no pain, no gain’, and were prepared for some mild discomfort to obtain some benefit with MET treatment; they thought that experiencing some muscle discomfort meant the intervention was ‘doing something’. One participant summed up his attitude to muscular discomfort during the MET session:

Yeah that happened a couple of times to me. I didn’t mind it … if it hurts, it’s doing something. (Male, focus group 3).

#### Perceptions of participating in the trial

This theme describes the perceptions of the different aspects of participating in the trial. Two subthemes emerged that included how the participant felt about the interactions they had with the researcher and their opinions on having to do spirometry.

##### Personnel

Participants shared their experiences of interacting with the researcher and how that made them feel during the trial. Participants said that they felt comfortable with the researcher and acknowledged various things the researcher did to make them feel more comfortable, such as having a pleasant disposition and providing a friendly environment for them. One participant described his experience with the researcher:

Because she would have a joke, or she’d say something funny, or she would laugh at something that you’d said, and you sort of get off the subject for a minute. But then she would go back, totally professional. So, the beauty of that was, it made us feel comfortable. We’re not getting belted around or anything. It was really, like comfortable. (Male, focus group 1).

Several participants explained that they were comfortable with the researcher and felt heard. They valued the clear and thorough explanations from the researcher prior to performing any movement, which helped participants know what to expect, understand the process and feel at ease:

We got what we could know, should know and the must know. That’s what we got. And I thought that was really good. That’s a sign of a good instructor. (Male, focus group 1).

And very respectful. She has always let me know—I’m sure she did to all of us—what she was about to do and where she was about to place her (hands) … and I felt very grateful for that information. Every time you did anything you just, you know … I knew what to expect. (Male, focus group 2).

In addition to sharing their experiences with the researcher, one participant shared their perception of being involved in the study and having access to multiple people as part of a management plan:

It’s sort of a holistic approach to the condition. We had the physiotherapists here to do our exercises, and they brought in a (respiratory) doctor, and staff gave lectures about the condition and treatments, and that sort of thing. So, they gave us the information there. Um, and they had one of the, ah, pharmacists come up and make sure we were using our puffers correctly to take our medicine, or whatever our treatments were. So, I felt that, ah, that [lead researcher’s] work was like another level; and apart from the benefits of her exercises and so forth, it was another person as a reference, or someone we could ask the questions of, and someone who was interested in how you were going. And so, I think as part of an overall thing there was great benefit in addition to, ah, hopefully from the exercise, the stretching. (Male, focus group 2).

##### Getting breathing right

A unique aspect of the trial was obtaining inspiratory capacity (IC) measurements before and after the MET sessions. Conducting the IC measurements required participants to stabilize their breathing before taking a big breath in, which some people found difficult to do. Discussion about participants’ experiences doing the IC tests often lead to a lot of laughter during the focus groups as participants recalled their attempts to ‘get it right’:

I also found that, ah, it took me a little while to sort of develop the technique. You know, controlling (my breathing), because otherwise I’d start off too hard, and by the time I was supposed to do the big one I’d run out of air (*laughing*). (Male, focus group 2).

Some people described a feeling of being given something to achieve—a target to reach in obtaining a reading for the IC. One person said that he felt ‘very anxious to reach that target’ while another person felt it was a ‘motivation’. Two others admitted that sometimes they felt foolish when they were unable to get a reading. Participants also compared the way they worked with the researcher to help them obtain the IC measurements:

So, [lead researcher] learnt with me ‘don’t look at it [computer]. Just look away, I’ll give you the prompts’ and then it worked better for me, and then it did work heaps better. (Female, focus group 3).

But I was the exact opposite. I had to look at it, look at the [computer] screen and then I was fine (*laughing).* (Male, focus group 3).

During the discussions, some participants said that they found it hard to breathe in deeply at the end of the test, as sometimes they felt puffed out. Interestingly, several participants felt that they had learnt a lot about their breathing from having to do the IC test:

And she taught me about the breathing, you know. The getting air, the problem with getting air out was a problem and I learnt a lot about that. It was really good. (Male, focus group 1).

#### Perceptions of the trial design

This theme describes aspects of the study that the participants liked and did not like about the study, and what they would change if given the opportunity. There were two subthemes that emerged that related to what the participant liked about the study in terms of the location, timing and number of MET sessions and suggestions for adding a home component.

##### Treatment features

A strategy to improve recruitment and compliance with attendance was to conduct the MET session at the same location as the PR, just prior to the start of the PR session. Participants generally felt that having the MET at the same location as the PR program made it easier for them to attend. One participant was happy to come to the hospital earlier for MET:

Well I don’t really have anything to lose, I am already coming in twice per week it doesn’t bother me coming in that half an hour early to give a little try. (Female, focus group 3).

All participants agreed that they would have liked to have more MET, whether that be an extra session per week or a longer duration of the session. Some participants said that they would have liked to do the MET session twice weekly, on the same days as PR, as they thought that would be more beneficial to them. The most common response from participants about what they would change about this study was to increase the duration of the MET sessions:

Timing? That’s the only one. Do it [MET] longer. (Male, focus group 3).

In addition to the suggestion of increasing the duration of the MET sessions, participants also commented on how much time they thought would be beneficial. The duration ranged from at least 30 min to 90 min. One participant remarked they would prefer to do MET by itself, instead of with PR:

I think most of us would’ve preferred just to have an hour and a half on that! (Male, focus group 2).

There were several participants who expressed concern over the cost of accessing a health professional who would be able to provide MET. Participants were cognizant of the costs of seeking allied health treatment privately and described their past experiences of seeing private allied health professionals for varying complaints, such as low back pain. One male was very clear about his reservations for accessing allied health outside of the trial:

The only thing is, I have a little bit against you (*gesturing to lead researcher*). It’s too bloody expensive. If I want some treatment with you people … It’s a hell of a lot expensive [sic], you know? (Male, focus group 1).

##### Wanting to do more

In addition to the discussion regarding cost to attend a private allied health professional, some participants delved into ways to make MET more accessible for everyone. Participants suggested a home component to the study, so that individuals could learn how to emulate the stretches they did within the study. One participant outlined his suggestion to reduce the cost for pensioners:

This MET … I felt good doing it … I would like to be able to use that to stretch, even in the gym, with all the equipment that they have there. And as a pensioner it doesn’t cost me too much, it’s affordable. Anything that you can do to help either work from home, or use basic gym equipment to do this MET, would be most gratefully accepted. (Male, focus group 2).

## Discussion

This novel study provides a comprehensive qualitative report of patient perceptions of adding a manual technique (MET) to routine care for people with moderate to severe COPD. The findings of this study provide key insights into the patient experience, including motivations for participating in the clinical study, participants perceived benefits of receiving MET and partaking in the study. Several participants described their experiences of living with COPD symptoms. They spoke about the anxiety they experience during both normal breathing as well as in times of increased work, causing breathlessness. In people with COPD, dyspnea (alongside cough, wheeze and chest tightness) has been shown to increase anxiety and depression and reduce quality of life [32–34]. The overlap of symptoms between dyspnea and anxiety confound the clinical presentation of a person with COPD [34].

The mechanism of dyspnea in COPD is multifactorial. It has been shown to limit exercise capacity and contributes to physical inactivity [35, 36]. As a subjective experience, the qualitative descriptors of breathlessness in COPD include perceived increased inspiratory effort and having a sensation of ‘unsatisfied inspiration’ [36, 37]. These sensations were reflected across the focus groups.

A major theme that transpired was that participants enjoyed partaking in the study and particularly enjoyed receiving the MET component. There were several factors that contributed to this, such as perceived physical benefits in relation to their COPD symptoms and also related to the timing with PR. Participants described feeling that it was easier to breathe and that they had more room to breathe both immediately after MET and after the trial. This is a similar finding to previous research that showed people with COPD felt their breathing was easier after receiving osteopathic technique [18, 19]. There were two participants that commented that they did not feel a change in their breathing, with one reasoning that it was because he had previously seen an osteopath for other musculoskeletal complaints. It is possible that there may be other factors that would affect participants’ perceptions of change in dyspnea in this trial, such as advancing disease and the compounding effects of comorbidities common to COPD on breathlessness (for example, unstable cardiac and renal disease).

Participants also reported that it felt good to stretch, and the muscle flexibility that the MET session allowed them was beneficial when going straight into the PR session. The term participants most often used was that they felt like MET was a good ‘warm up’ for going into the gym afterwards. Furthermore, some participants reported feeling that they were better able to partake in the PR exercises on the days they also had MET. In addition to the sensation of improved breathing immediately after the MET session, the confidence that participants felt in being ‘warmed up’ may also provide motivation to exercise. This is an interesting finding, given that an individual’s perception of dyspnea affects their motivations and likelihood of undertaking exercise [35, 38].

Additionally, participants saw value in an additional health professional in the management paradigm. The role of the researcher for the participants was viewed as an extra person to talk to about their health while they received MET at the same time. Participants also felt that they could trust the researcher and felt a sense of comfort around her. Clinician empathy has been shown to improve patient satisfaction and clinical outcomes [39, 40]. This highlights the importance of clinical empathy as a person-focused characteristic of healthcare professionals, one which is generated through trust between the patient and practitioner [41, 42]. PR staff observed that participants looked forward to seeing the researcher for MET, which resulted in improved attendance at PR (paper forthcoming).

Patient enablement and empowerment is another important factor in improving outcomes in chronic diseases [43]. For people with COPD, feelings of being in control of their own symptoms may be just as important as actual behaviors, such as attempting to increase exercise [44]. Participants in the present study reported that they had learned about aspects of their breathing from the researcher and were able to adapt their behaviors outside of the hospital. For example, participants discussed trying to emulate the MET stretches at home to help themselves feel more flexible and to help their breathing.

It is recognized that there are discrepancies between patient and clinician preferences in the management of COPD [8]. Awareness of the patient’s care preferences is important to understand how it might affect adherence or ‘buy-in’ to their own management strategies. This may lead to improvements in outcomes such as quality of life and prognosis. While participants perceived various benefits from the intervention, there was also concern about not being able to afford such treatments if they sought treatment privately. As such, the costs of complementary interventions must be considered by both patients and clinicians. Finally, providing a setting whereby the individual can gain confidence in their ability to control their COPD symptoms may decrease the intensity of the symptoms they experience [44].

### Strengths and limitations

This study provided novel information about clinical study participant’s experiences and perceptions of receiving a manual therapy technique alongside PR, and informs the feasibility of implementing a manual therapy trial alongside PR. Several limitations of this study are acknowledged. The use of convenience sampling in this study potentiates volunteer bias—a type of selection bias that occurs when volunteers from a sample may have different outcomes or perceptions of the study’s relevance compared to non-volunteers [45]. It is not possible to determine the views of non-participants as they declined participation. However, all clinical study participants who completed at least two MET sessions (considered ‘completers’) received a follow-up phone call asking about their experience with MET (27 of the 33 study participants; data not presented). All participants expressed that they enjoyed the MET treatment. This suggests that the views of participants who declined participation in focus groups were similar to those who participated, reducing the likelihood of volunteer bias.

Focus groups were conducted on site, which may have introduced the possibility of social desirability bias from participants. This type of bias refers to the tendency of an individual to present views that are thought to be socially acceptable, though may not truly reflect their honest account of the situation [46]. The researcher was on site while the focus groups were conducted to coordinate the delivery of the interviews, which may have affected the homogeneity in positive responses in the present study. However, this was minimized by triangulating data collection [47] and by the facilitator being able to clarify participant responses during face-to-face interviews [48].

The time between the end of the study for some participants and the focus groups may have affected recruitment rates and participants’ ability to recall past events. Notably, only three of the 15 clinical study participants (20%), who completed the clinical study before focus groups were introduced, agreed to participate in the focus groups; whereas nine of the remaining 12 eligible participants (75%) participated after focus groups were introduced. There were several reasons for declining participation in the focus groups; these included moving interstate, having other appointments on the days of the focus groups and being unwell. The potential for recall bias was managed in several ways, including familiarity with the researcher who conducted the phone calls to eligible participants, the use of a semi-structured interview guide with prompts to increase recall of the logistical elements of the trial, and an experienced facilitator to build rapport to elicit truthful answers.

Finally, focus groups were organized with at least six participants; however, in each focus group, at least one participant was unable to attend the appointment due to unavoidable reasons, such as being unwell. Ultimately, this led to focus group sample sizes that were less than the optimal number of 6–10 [27, 28]. Thus, the findings may not be generalizable to other settings and for people with milder disease.

### Implications for clinical research and practice

There are several implications that have been gained from this study for both future research and clinical practice. First, the patient lived experience with COPD is one that can be both a barrier and a motivator to participating in a PR program. Knowing an individual’s barriers and motivators for changing behavior in COPD is paramount to patient-centered care.

One of the major goals in the management of COPD is to increase the amount of daily activity that an individual performs, which will directly relate to improvements in exercise capacity and both resting and exertional dyspnea [49]. MET may be an appropriate adjunctive therapy for people with moderate to severe COPD, as participants reported benefits such as improved ability to breathe, improved muscular flexibility and an improved feeling of being warmed up for PR. Furthermore, the MET component of the study was enjoyed by every participant who was interviewed, although participants would have preferred more frequent treatment and mild discomforts were noted.

It is possible that the positive experiences and perceptions reported by participants may have occurred because the study was conducted in a research setting. If such a service were implemented alongside PR as part of standard patient care, there may be similar challenges to those faced by PR, such as increased numbers of patients with less time able to be allocated to individuals.

The Global Initiative for Chronic Obstructive Lung Disease (GOLD) guidelines recommend a patient-centered approach for the management of COPD, one which aims to modify the behavior of individuals and motivate physical activity [3]. The participant experience of receiving MET in addition to PR should be taken into consideration when developing multidisciplinary management strategies for people with moderate to severe COPD. The perceived benefits reported by participants, coupled with high attendance rates to MET, cannot be overlooked. Additionally, due to the aforementioned benefits of the implementation of MET within the clinical study, the methodology may be utilized in future clinical trials.

## Conclusion

People with moderate to severe COPD enjoyed receiving MET alongside a PR program. Participants also reported perceived benefits from MET, such as feeling like they were better prepared for PR, had more flexibility and found it easier to breathe. There were a small number of participants who experienced mild musculoskeletal discomfort in relation to the MET treatment. This study provides initial evidence of the potential role of adjunctive manual therapy in the multidisciplinary management of COPD. The overwhelmingly positive response in relation to receiving the MET treatment in conjunction with PR, coupled with the anecdotal improvement in attendance to PR after MET, shows that this form of adjunctive therapy may play an important role in the patient-centered management of people with moderate to severe COPD.

## Supplementary Information

**Additional file 1.** Patient position during MET (a) seated and (b) side-lying. Description: This document presents two images of typical patient positions used for MET application during the study.

**Additional file 2.** COREQ (COnsolidated criteria for REporting Qualitative research) Checklist. Description: This document states where in the manuscript the COREQ items have been reported.

**Additional file 3.** Participant Interview Guide. Description: This document lists the questions asked of participants during focus groups.

## Data Availability

The data that support the findings of this study are available from the corresponding author, MC, upon reasonable request.
