# Supplementary figures and images for: Perceptions and experiences of a manual therapy trial: a qualitative study of people with moderate to severe COPD
Source: Chiropr Man Therap. 2021 Jul 27;29:27. doi: 10.1186/s12998-021-00387-0 (PMC8314605; doi:10.1186/s12998-021-00387-0)

## Additional File 1

**Patient position during MET (a) seated and (b) side-lying.**

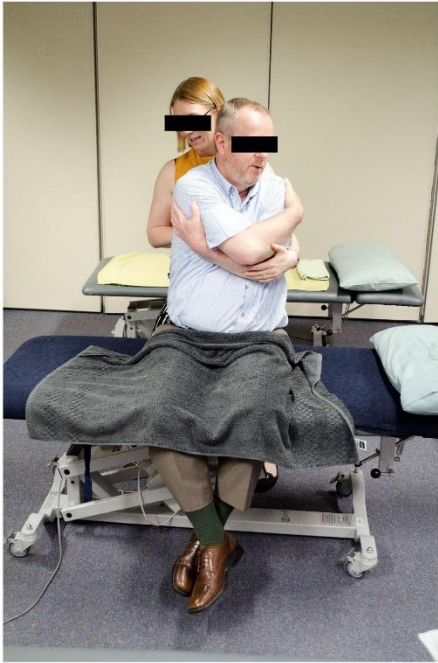

(a)

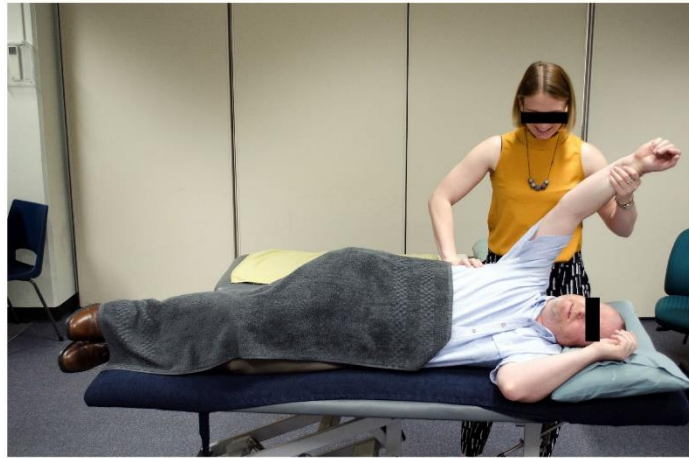

(b)

Supplement: Supplementary file 1 — Additional file 1. Patient position during MET (a) seated and (b) side-lying. Description: This document presents two images of typical patient positions used for MET application during the study. [file 12998_2021_387_MOESM1_ESM.pdf]
