# Supplementary material for: Perceptions and experiences of a manual therapy trial: a qualitative study of people with moderate to severe COPD
Source: Chiropr Man Therap. 2021 Jul 27;29:27. doi: 10.1186/s12998-021-00387-0 (PMC8314605; doi:10.1186/s12998-021-00387-0)
Supplement: Supplementary file 3 — Additional file 3. Participant Interview Guide. Description: This document lists the questions asked of participants during focus groups. [file 12998_2021_387_MOESM3_ESM.docx]

Additional File 3

Participant Interview Guide

The questions below are examples of questions which may be used to guide focus groups with participants. The order of questioning may vary depending on participants’ responses, and may be subject to minor revisions throughout the duration of the project as new information and themes are identified.

1. If you think back to when you received the information about the Muscle Energy Technique study, can you remember why you decided to join this study?
2. What were you expecting the muscle stretching treatment to be like?
3. Was the treatment what you were expecting? Why or why not?
4. Can you tell me about your experience of receiving muscle stretching treatment?
5. Some people may feel a bit of muscle soreness after the treatment. Did you notice this or any other changes after treatment?
6. Can you tell me about any other changes that you noticed in your daily activities in relation to your COPD?
7. Now I’m going to ask some questions about the way the study was run. The muscle stretching treatments usually lasted for about 15 minutes. Do you think this was this too short, too long or about right?
8. If the study had involved muscle stretching treatments twice weekly, do you think you would have still participated in the study?
9. When you came in for treatment, you had to do the breathing test before and after treatment. What was your experience in doing the breathing test after the treatment?
10. Do you think the timing of the treatment being just before your pulmonary rehabilitation helped or didn’t help? Why?
11. What did you like about participating in the study?
12. What didn’t you like about participating in the study?
13. If you had the ability to change one thing about the study, what would you change?
14. We’ve covered quite a few topics here, is there anything else you’d like to add?
